# Exploring the Relationship Between Sleep Quality, Sleep-Related Biomarkers, and Motor Skill Acquisition Using Virtual Reality in People With Parkinson's Disease: A Pilot Study

**DOI:** 10.3389/fneur.2021.582611

**Published:** 2021-03-02

**Authors:** Alham Al-Sharman, Ismail Alhaj Ismaiel, Hanan Khalil, Khalid El-Salem

**Affiliations:** ^1^Department of Rehabilitation Sciences, Faculty of Applied Medical Sciences, Jordan University of Science and Technology, Irbid, Jordan; ^2^Department of Neurosciences, Faculty of Medicine, Jordan University of Science and Technology, Irbid, Jordan

**Keywords:** Parkinson's disease, motor learning, sleep, rehabilitation, virtual reality

## Abstract

**Background and Objectives:** Despite the fact that sleep disturbances are among the most common and disabling manifestations of Parkinson's disease (PD), no study has investigated the effect of sleep quality and sleep-related biomarkers on motor skill acquisition in people with Parkinson's disease (PwPD).

**Objective:** To examine the relationship between skill acquisition, sleep quality, and sleep-related biomarkers in PwPD using virtual reality (VR) system.

**Methods:** This is a cross sectional study conducted on 31 PwPD and 31 healthy controls. To assess skill acquisition, each participant practiced a VR game 6 times (blocks). The main outcomes from the VR game were the required time to complete the VR game and the recorded errors. Motor skill acquisition was calculated as the difference of scores between block 6 and block 2 for both outcomes. Sleep was assessed subjectively using Pittsburgh Sleep Quality Index (PSQI) and objectively using the Actisleep. To assess sleep related biomarker, plasma serotonin level was examined.

**Results:** PwPD and healthy controls demonstrated a practice-related improvement in performance as shown by the main effect of block for each of the VR outcome measures (*p* < 0.000, time required to complete VR game; *p* < 0.000, recorded errors). There was no interaction effect between Block X Group for both outcome measures. There were significant correlations in both groups (*p* < 0.05) between motor skill acquisition (as indicated by the difference of time required to complete the VR game between block 6 and block 2) and PSQI total score, wake after sleep onset, and sleep efficiency. Additionally, a significant correlation was observed in both groups between motor skill acquisition (as indicated by the difference of time required to complete the VR game between block 6 and block 2) and the plasma serotonin level (*p* < 0.05). These correlations in PwPD remained significant, even after adjusting for disease motor severity, cognitive status, depression, and daily dose of L-dopa.

**Discussion and Conclusions:** Sleep quality may influence motor skill acquisition in PwPD. Healthcare professionals are encouraged to be aware about sleep quality and sleep assessment tools. Therapies may target improving sleep quality which could result in improving motor skill acquisition.

## Introduction

The loss of dopaminergic cells in Parkinson's disease (PD) causes the cardinal motor symptoms of PD and contributes to non-motor symptoms, including autonomic dysfunction, cognitive impairments, and sleep disturbances (1, 2). Currently pharmacological and surgical treatment options are not always effective in managing common motor and non-motor symptoms associated with PD (3, 4). Non-pharmacological rehabilitation treatment options such as physical therapy showed effectiveness and are considered to be important in the journey of managing People with Parkinson's disease (PwPD) (5). Physical therapists play a major role in rehabilitating PwPD by planning activity-focused interventions which emphasize the need for practice and repetition of purposeful motor actions in challenging environments. However, research has indicated that PD would lead to degradation of motor skill learning (6). Therefore, PwPD often have difficulty acquiring and learning new motor tasks affecting therapy outcomes (7, 8). Specifically, studies have demonstrated that different aspects of motor skill learning (i.e., skill acquisition, consolidation, retention, and transfer) are more impacted among PwPD compared to healthy individuals (8).

Overall, the etiology underlying the deficits in motor skill learning in PD is not very clear. However, it is likely to be multi-factorial. For example, the impairments in basal ganglia and reduced dopaminergic neurotransmitter can be a main cause (9). Degeneration in striatum has been linked to deficits in learning motor sequences in PD particularly the consolidation phase (10). Studies have also reported a number of factors related to PD that might have a major impact on motor skill learning such as severity and disease duration (6, 11). In addition, non-motor symptoms including cognitive deficits have been found to affect motor skill learning in PwPD (12).

Recently, there is a growing attention toward the role of sleep on motor skill learning. Sleep may impact each stage of motor skill learning. Good sleep quality was found to enhance the consolidation phase of motor skill learning offline (i.e., when no practice is occurring; “sleep-dependent off-line motor learning”) across a wide range of motor tasks in healthy adults as well as in some neurological conditions such as stroke (13). After a night of 8 h sleep, performance on simple motor tasks improved in comparison to same time wakefulness (14). Furthermore, few recent studies found that lower levels of sleep quality before learning the motor task, negatively impacted motor skill acquisition in young healthy adults (14) and in people with sleep disorders (15).

Around more than 3 quarters of PwPD have sleep disorders (16). Excessive daytime sleepiness, REM sleep behavior disorder, and fragmented sleep are frequently reported in this population (17). As yet, there is little knowledge about the role of sleep on motor skill learning in PwPD (18). Terpening et al. (18) demonstrated that sleep is important in the consolidation phase of motor learning. However, this later study did not examine the effect of sleep quality on motor skill acquisition in PwPD. In motor skill learning, successful acquisition results in attaining a certain level of task ability which leads to rapid formation of a memory representation within the brain. Generally, task-related activations in a motor-related network during the initial learning session predicted subsequent consolidation changes in motor behavior (19). Therefore, motor skill acquisition is important stage of motor skill learning and further investigations to understand the impact of sleep on motor skill acquisition in PwPD is warranted.

When assessing motor learning in general, it is important to consider the task under investigation. For example, the study by Terpening et al. (18) used a simple motor task (i.e., finger tapping) to understand motor skill learning in PwPD. However, most daily tasks are more functional and complex. A review by Wulf and Shea (20) state that learning complex motor tasks requires high motor demands, fast reaction to environmental stimuli, and different body parts coordination. Therefore, there is a need to understand motor skill acquisition in PwPD using complex tasks that are functional and simulate tasks similar to real life and rehabilitation settings.

Another aspect of sleep quality that might affect motor skill learning is the role of the hormone serotonin on motor skill acquisition. Previous studies showed low levels of serotonin transporter in parkinsonism within the striatal area (21) and that up to 50% of PwPD have decreased levels of serotonin (22). A recent investigation found significant correlation between reduction of serotonin in midbrain, basal ganglia and hypothalamus, and sleep disturbances in PwPD (23). Furthermore, acute increases in serotonergic transmission could influence skill acquisition during motor learning (24). Therefore, the aims of this study were to: (1) examine motor skill acquisition in PwPD compared to age and gender matched healthy controls using a functional motor task similar to real life, (2) examine the relationship between motor skill acquisition and sleep quality in PwPD, and (3) examine the relationship between plasma serotonin level and motor skill acquisition in PwPD.

## Materials and Methods

### Study Design and Participants

This cross-sectional study was designed to examine motor skill acquisition in PwPD compared to age and gender matched healthy controls using a functional motor task and also to examine the relationship between sleep quality, sleep-related biomarker, and motor skill acquisition in PwPD. Thirty-one PD participants and 31 age and gender matched healthy controls were recruited into the study. PD participants were recruited from King Abdulla University Hospital (KAUH) and additionally from a research database of Jordan University of Science and Technology (JUST). PwPD were screened for eligibility by a neurology consultant at KAUH; who is responsible for their care. Eligible subjects were invited to participate in the study. Inclusion criteria were: (1) a neurologist-confirmed diagnosis of idiopathic PD, (2) capacity to give informed consent, (3) modified Hoehn and Yahr Stage 1–4 during the “ON stage” of medication, (4) maintaining a stable medical regime for 3 weeks prior to initiation of study, and (5) a participant, who had no experience with the motor task implied in this study but still physically able to perform it without physical assistance. Exclusion criteria were: (1) presence of additional neurological disorders that may affect balance and gait (e.g., head injury, stroke, vestibular dysfunction, or peripheral neuropathy), and (2) the presence of severe cognitive deficits or behavioral disorders preventing safe participation.

Healthy controls were recruited from local community and friends of people with PD introduced to us by the patient. Inclusion criteria of the healthy-control participants included: (1) age and gender matched individuals, (2) being functionally independent. Participants were excluded if they had reported (3) untreated sleep disorders, including sleep apnea (4) a history of neurological disorders; and any orthopedic problems or mobility deficits that prevented them from performing the study task (5) taking any medications that may affect sleep and serotonin level.

Participants gave a written informed consent approved from the Institutional Research Committees of Jordan University of Science and Technology (ID: JUST-AA-2018-525).

### Study Procedure

Each participant was asked to visit the physical therapy laboratory at Jordan Science and Technology University (JUST) for a single assessment session. At the beginning of the session, blood samples were collected from the participants and stored for later analysis for determining the plasma serotonin levels. In order to eliminate the possible effect of dopaminergic medication on the plasma serotonin levels, fasting blood samples were taken in the early morning (i.e., at 8:00 A.M. ±1 h) after overnight withdrawal of the medication (during the “OFF” phase). Following this, participants took their regularly scheduled morning dose of medication and were provided with a resting period until they were notably on their “ON” stage in which assessments for motor skill acquisition, subjective sleep quality, and cognitive status were undertaken. Collection of basic personal and demographic information such as age, gender, daily use of L-dopa, and disease severity were also obtained from each participant. A sub sample of the PD participants (*n* = 22) were asked to wear an Actisleep (ActiGraph; Pensacola, FL) device a week before the testing session to objectively asses sleep quality (see details below).

### Motor Task Description

**Figure 1:**
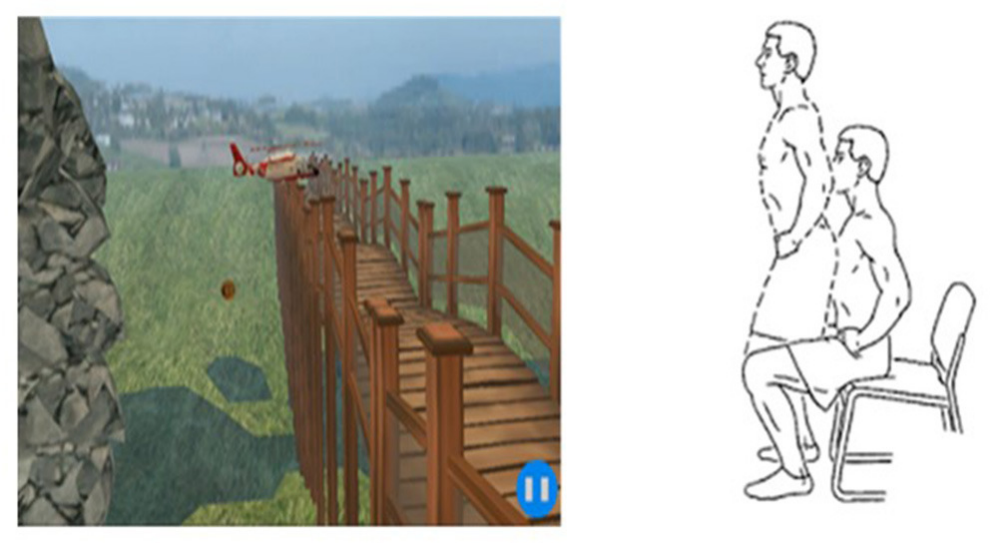
Sit to stand game through virtual reality.

To assess motor skill acquisition, participants were asked to perform a novel virtual reality (VR) game during their “ON” stage. The novel VR game was developed as part of a previously conducted study (25) and has been validated as an assessment and treatment tool (26). The VR game is part of a non-immersive VR system developed by our research team, which consists of the Microsoft Kinect sensor, large standard LCD monitor, and its software on a research laptop device. Details are published elsewhere (25). In brief, in the VR game, participants were asked to steer a helicopter up and down to collect coins and to avoid specific number of obstacles by moving from chair (the chair is without arm) from sitting to standing and vice versa (Figure 1). The aim of the game is to collect as many coins as possible while avoiding specific number of obstacles at the same time by performing the sit to stand movement. The numbers of missing coins (recorded errors) and the time that required to go through the obstacles by the participants determined the game score which was given by the system itself.

Each participant performed VR game for 6 times (blocks) during the session. In order to familiarize the participants with the game, the first block was performed and discarded from analysis. To prevent fatigue, participants were allowed to rest between blocks if needed (in sitting position). In this study, required time to complete the game and errors were recorded from the virtual game for each block performed by the participants. Difference of scores between block 6 and block 2 for both outcomes (i.e., time to complete the block and recorded errors) were considered to represent motor skill acquisition and training-related gains in performance.

### Sleep Quality Measures

The Arabic version of the Pittsburgh Sleep Quality Index (PSQI) was used to subjectively assess sleep quality (27). The PSQI is a well-validated and reliable measure of sleep quality which consists of 19 self-rated questions forming a global score ranging from 0 to 21 (28). A global score of 5 or more reflects poor sleep quality for all age groups (28). The PSQI as a generic measure of sleep quality was commonly used in PwPD (29).

To objectively assess sleep quality, we utilized the Actisleep. Only a sub-sample of PD participants (*n* = 22) wore the Actisleep due to the limited number of Actisleep devices available for this study and the limited time allowed to complete this study. Participants were asked to wear an Actisleep (Actigraph wGT3X-BT, Pensacola, Florida, USA) device for 7 consecutive nights until the day the participants came in for the motor skill acquisition testing session. Participants were asked to wear the Actisleep around the non-dominant wrists while maintaining normal lifestyle, especially sleeping habits. Actisleep (30) is a tri-axial accelerometer developed to measure sleep/wake positions in which the following sleep parameters were calculated: sleep efficiency (SE; number of sleep minutes divided by the total minutes the subject was in bed), and wake after sleep onset (WASO; time of wake in minutes after sleep onset). Actisleep was found to be a valid and reliable device for sleep measurement among healthy young adults (31). The clinical utility for using the Actisleep with PD individuals has been proven in several studies (32). Actisleep signals were sampled at 30 Hz. Data from Actisleep was analyzed using Actilife software (ActiGraph; Pensacola, FL) (31).

### Other Outcome Measures

To account for confounding variables; data regarding disease motor severity, cognitive status, depression, and anxiety of the participants were recorded. Disease motor severity was assessed using the Movement Disorder Society-Unified Parkinson's Disease Rating Scale (MDS-UPDRS)-Part III (33), as well as the Hoehn-Yahr staging system (34). Cognitive status of the participants was evaluated using the Arabic version of the Montreal Cognitive Assessment (MOCA) (35); the total score of the MOCA was used in the analysis. The Arabic version of the Hospital Anxiety and Depression Scale (HADS) was used to evaluate the level depression (36, 37). L-dopa daily dose as well as personal data including age and gender were also collected. All data was recorded in the morning during the “ON” state.

### Sleep Related Biomarkers

Fasting blood samples were collected for measurement of plasma serotonin from the PD participants at 8:00 A.M. ±1 h. Plasma serotonin level was examined using the sandwich enzyme-sorbent assay technology (38). After blood collection, all blood samples were centrifuged at 1,500 × g for 15 min in order to collect plasma. Following this, plasma was stored at −80°C until used. A competitive Serotonin/5 hydroxytryptamine (5-HT) ELIZA kits for quantitative analysis of total plasma of serotonin was used (abx257126). All assays were performed according to the instructions provided by the manufacturer Abbexa, Cabridge, UK.

### Statistical Analysis

Statistical analyses were performed with Statistical Package for the Social Sciences software (SPSS 20.00). Independent- sample *t*-tests were used to assess differences in participants' characteristics between groups. Also, independent- sample *t*-tests were used to assess the differences between participants who wore the Actisleep and those who did not wear it considering the characteristics that might affect the results including the severity of motor symptoms (represented by the MDS-UPDRS Part III) and age. Performance through blocks was examined using a two-factor [Group (PD, healthy control) × Block (2, 3, 4, 5, 6)] repeated measures ANOVAs with time to complete VR game and number of errors recorded were considered the dependent variables. *Post-hoc* analysis was conducted using LSD for multiple comparison between blocks. In all comparisons, significance level was set at 0.05.

Motor skill acquisition was calculated as the differences in performance between Block 6 and Block 2 for time to complete VR game and recorded errors. The associations between subjective and objective sleep measures, sleep biomarker and motor skill acquisition were assessed for both groups using Pearson correlation coefficient (*r*). In general, *r* > 0.50 indicates large correlation, 0.31–0.49 indicates moderate correlation, and <0.30 indicates poor correlation (39). To account for confounding factors including the severity of motor symptoms (represented by the MDS-UPDRS Part III), the cognitive level of the participants (represented by the MOCA total score), and depression (represented by HADS depression score) and the daily dose of L-dopa, the relationship between sleep measures, sleep biomarker, and motor skill acquisition was examined using partial correlation analysis.

## Results

### Subject Characteristics

Table 1 demonstrates the demographic and clinical data for both groups (i.e., PwPD and healthy controls). There were no significant differences between groups in term of age (*p* = 0.09) and HADS depression (*p* = 0.19). Significant differences between groups were observed in the PSQI (*p* = 0.04), MOCA (*p* < 0.001), and plasma serotonin level (*p* = 0.01). There were no significant differences in age and MDS-UPDRS Part III between participants who wore the Actisleep and those who did not wear it (*p* > 0.05).

**Table 1:** Demographic and clinical data of participants in both groups.

**Parameters**	***N***	**PwPD**			**Healthy controls**		***P*-value** **(between groups)**
	***N***	**Mean**	**SD**	**N**	**Mean**	**SD**	
Gender (F/M)	31	10/21	–	31	10/21	–	–
Age (years)	31	61.06	7.66		57.42	8.98	0.09
MOCA total score (unit)	31	19.89	4.22	31	22.97	3.5	<0.001
HADS Depression	31	7.6	6.1	31	5.7	4.2	0.19
PSQI total score (unit)	31	8.48	4.03	31	5.32	3.32	0.04
		PSQI <5: (21)PSQI > 5: (10)			PSQI <5: (16) PSQI > 5: (16)		
WASO	22	15.48	10.75	–	–	–	–
Sleep efficiency (%)	22	69.20	19.66	–	–	–	–
Plasma serotonin level (pg/ml)	31	119.6	24.63	31	177.47	22.7	0.01
MDS-UPDRS- Part III (unit)	31	36.66	12.67	–	–	–	–
Hoehn & Yahr (HY) (unit)	31						
	5	Stage 1		–	–	–	–
	16	Stage 2					
	9	Stage 3					
	1	Stage 4					
Daily dose of L-dopa	31	698.2	262.02	–	–	–	–

*MOCA, Montréal Cognitive Assessment; HADS, Hospital Anxiety and Depression Scale; PSQI, Pittsburgh Sleep Quality Index; WASO, Average awaking time after sleep onset (min) deriving from Actisleep. Sleep efficiency, number of sleep minutes divided by the total minutes the subject was in bed deriving from Actisleep. MDS-UPDRS, Movement Disorder Society-Unified Parkinson's Disease Rating Scale*.

### Motor Skill Performance

**Figure 2:**
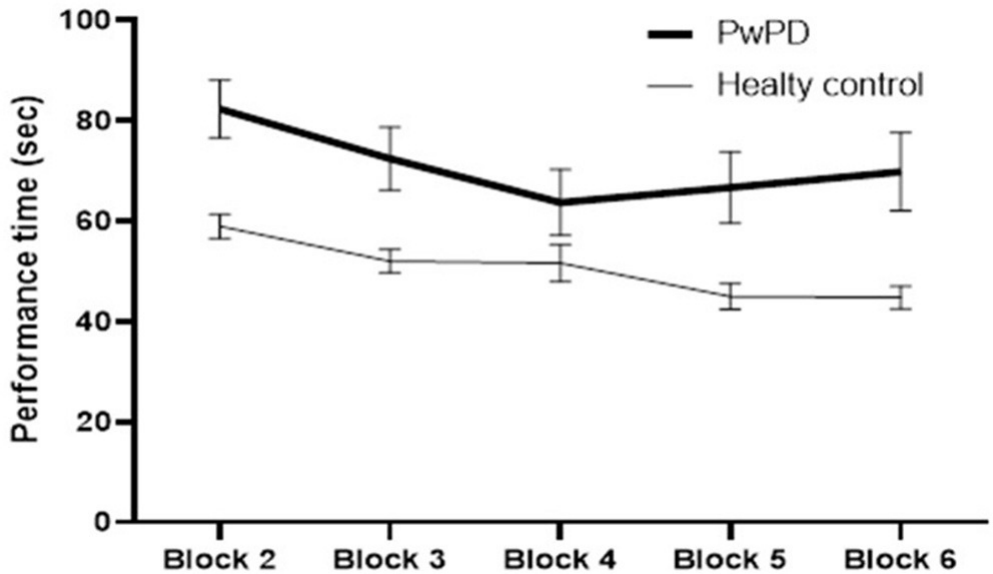
Practice-related improvement in performance (time required to complete the VR game in seconds) across blocks in PwPD and healthy controls. *Post-hoc* analysis indicated significant difference between Block 6 and Block 2 in both groups. In healthy controls, significant differences were also found between (Block 2 and Block 3) and between (Block 4 and Block 5). In PwPD, significant differences were also found between (Block 2 and Block 3) and between (Block 3 and Block 4).

**Figure 3:**
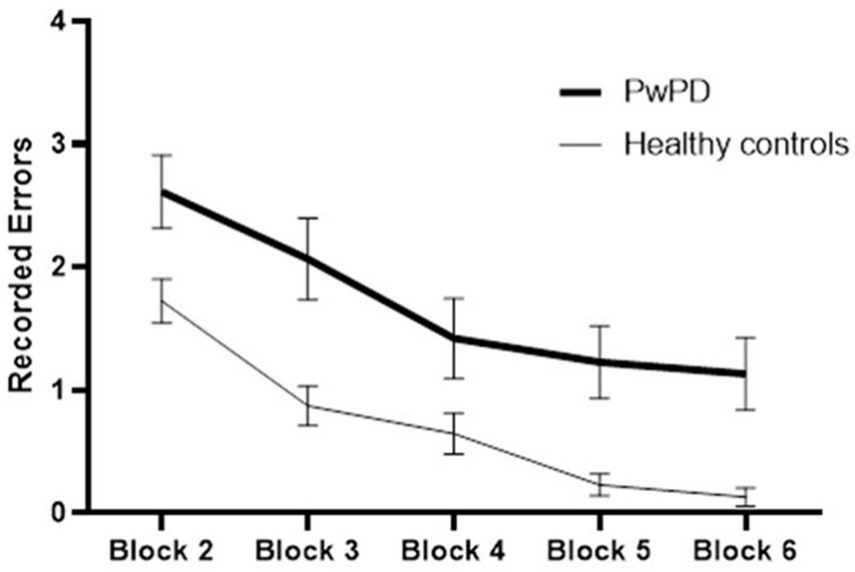
Practice-related improvement in performance (recorded errors) across blocks in PwPD and healthy controls. *Post-hoc* analysis indicated significant difference between Block 6 and Block 2 in both groups. In healthy controls, significant differences were also found between (Block 2 and Block 3) and between (Block 3 and Block 4). In PwPD, significant differences were also found between (Block 4 and Block 5), between (Block 3 and Block 4), and between (Block 3 and Block 4).

The participants in both groups demonstrated a practice-related improvement in performance, as shown by the main effect of block for each of the outcome measures (*p* < 0.000, time required to complete VR game; *p* < 0.000, errors recorded) (Figures 2, 3). The extent of improvement in performance across blocks revealed significant differences between PwPD and healthy control in motor skill acquisition as indicated by the main effect of group for time required to complete VR game (*p* = 0.04) and errors recorded (*p* = 0.01). There was no interaction effect between Block X Group for both outcome measures (time to complete VR game *p* = 0.31; errors recorded, *p* = 0.42).

### Correlations Between Motor Skill Acquisition, Serotonin, and Sleep Measures

Quality of sleep, as indicated by PSQI, was significantly associated with plasma serotonin level for both groups (*r* = −0.519, *p* = 0.003, for healthy controls; *r* = −0.48, *p* = 0.006, for PwPD). Poor quality of sleep was significantly associated with lower plasma serotonin level in both groups.

Table 2 summarizes the differences between Block 6 and 2 for both outcome measures (time required to complete VR game and recorded errors) which represent motor skill acquisition for PwPD and healthy control. Also Table 2 presents the results of *post-hoc* analysis between Block 2 and Block 6. The results indicate significant performance changes between blocks 2 and block 6 in healthy controls and PwPD in both outcome measures.

**Table 2:** Motor skill acquisition in PwPD and healthy controls and *Post-hoc* analysis (Pairwise comparison) between Block 2 and Block 6.

**Outcome measures**	**PwPD**				**Healthy controls**			
	**Block 2**	**Block 6**	**Motor skill** **acquisition**	**Pairwise comparison** **Sig**.	**Block 2**	**Block 6**	**Motor skill** **acquisition**	**Pairwise comparison** **Sig**.
Performance time	83.33 (32.16)	68.82 (43.2)	−12.5 (35.6)	*p* = 0.001	58.9 (13.3)	44.8 (12.6)	−14.18 (18.2)	*p* = 0.05
Recorded Errors	2.61 (1.7)	1.13 (1.6)	−1.48 (1.7)	*p* <0.001	1.72 (0.9)	0.13 (0.4)	−1.6 (1.0)	*p* <0.001

*Motor skill acquisition was calculated as the differences between Block 6 from Block 2 for both outcome measures. Negative motor skill acquisition for recorded errors and performance time in both groups indicate better performance in Block 6 in relative to Block 2*.

Table 3 summarizes the relationship between motor skill acquisition, sleep quality measures, and plasma serotonin in PwPD and healthy controls. In healthy controls, there was significant correlation between motor skill acquisition (as indicated by the difference of time required to complete the VR game between block 6 and block 2) and PSQI total score. Additionally, a significant correlation was observed between motor skill acquisition (as indicated by the difference of time required to complete the VR game between block 6 and block 2) and the plasma serotonin level (*p* < 0.05). These correlations between motor skill acquisition, PSQI total score, and plasma serotonin level in healthy controls remained significant, even after adjusting for cognitive status and depression (Table 4).

**Table 3:** Correlations between motor skill acquisitions and sleep quality measures.in the PD and healthy control participants.

**Sleep quality** **measures**	**PwPD**		**Healthy control**	
	**Performance** **time**	**Error** **recorded**	**Performance** **time**	**Error** **recorded**
PSQI total score	*r* = 0.64 *p* = 0.0001	*r* = 0.06 *p* = 0.74	*r* = 0.71 *p* <0.001	*r* = 0.17 *p* = 0.36
Sleep efficiency	*r* = −0.66 *p* = 0.001	*r* = −0.13 *p* =0 0.54	——————	——————
WASO	*r* = 0.66 *p* = 0.001	*r* = 0.25 *p* = 0.27	——————	——————
Serotonin level	*r* = −0.48 *p* = 0.006	*r* = −0.15 *p* = 0.42	*r* = −0.63 *p* = <0.001	*r* = −0.11 *p* = 0.56

*Performance time represents motor skill acquisition outcome which is calculated as the change in the time required to complete the VR task between block 6 and 2. Recorded error represents motor skill acquisition outcome which is calculated as the difference in the error recorded during the VR task between block 6 and 2. PSQI, Pittsburg Sleep Quality Index; WASO, Average awaking time after sleep onset (min) deriving from Actisleep. Sleep efficiency: number of sleep minutes divided by the total minutes the subject was in bed deriving from Actisleep*.

**Table 4:** Partial correlation in healthy controls between motor skill acquisition outcomes and sleep quality measures and plasma levels of serotonin controlling cognitive status and depression level.

		**Plasma serotonin** **level**	**PSQI total** **score**
Performance time	Correlation coefficient	−0.62	0.72
	*P*-value	0.02	0.03
Recorded error	Correlation coefficient	0.15	025
	*P*-value	0.48	0.26

*Control variables, Montréal Cognitive Assessment (MOCA) total score and Hospital Anxiety and Depression Scale. Performance time represents motor skill acquisition outcome which is calculated as the change in the time required to complete the VR task between block 6 and 2. Recorded error represents motor skill acquisition outcome which is calculated as the difference in the error recorded during the VR task between block 6 and 2. PSQI, Pittsburg Sleep Quality Index*.

For PwPD, there were significant correlations (*p* < 0.05) between motor skill acquisition (as indicated by the difference of time required to complete the VR game between block 6 and block 2) and PSQI total score, wake after sleep onset in minutes (WASO; i.e., the total amount of time the participants spent awake after falling asleep), and sleep efficiency. Additionally, a significant correlation was observed between motor skill acquisition (as indicated by the difference of time required to complete the VR game between block 6 and block 2) and the plasma serotonin level (*p* < 0.05) (Table 3). These correlations between motor skill acquisition and plasma serotonin level, PSQI total score, average awaking time, and sleep efficiency in the PD participants remained significant, even after adjusting for disease motor severity, cognitive status, and daily dose of L-dopa (Table 5).

**Table 5:** Partial correlation in PD participants between motor skill acquisition outcomes and sleep quality measures and plasma levels of serotonin controlling for disease motor severity, cognitive status, daily dose of L-dopa, and depression level.

		**Plasma** **serotonin** **level**	**PSQI** **total** **score**	**Sleep** **efficiency**	**Avg. wakening** **time after** **sleep onset**
Performance time	Correlation coefficient	−0.59	0.66	−0.55	0.62
	*P*-value	0.04	0.01	0.05	0.02
Recorded error	Correlation coefficient	−0.44	−0.08	0.1	0.3
	*P*-value	0.1	0.8	0.8	0.3

*Control variables: Movement Disorders Society Unified Parkinson's Disease Rating Scale (MDS-UPDRS) Part III, Montréal Cognitive Assessment (MOCA) total score, Hospital Anxiety and Depression Scale and daily dose of L-dopa. Performance time represents motor skill acquisition outcome which is calculated as the change in the time required to complete the VR task between block 6 and 2. Recorded error represents motor skill acquisition outcome which is calculated as the difference in the error recorded during the VR task between block 6 and 2. PSQI, Pittsburg Sleep Quality Index*.

On the other hand, no significant correlations were noted between the motor skill acquisition as indicated by recorded errors and any of the sleep quality measures nor the plasma serotonin level in both groups.

## Discussion

To our knowledge, this is the first study to investigate the relationship between subjective and objective sleep quality measures and motor skill acquisition in PwPD. What further makes this study important, is the novel investigation of the relationship between a sleep-related biomarker (i.e., serotonin) and motor skill acquisition in PwPD. The results demonstrated significant associations between subjective (PSQI) and objective (Actisleep measures) sleep measures and the acquisition of the VR game even after controlling for the L-dopa use, disease motor severity and cognitive status. Furthermore, we found that lower plasma serotonin level is significantly associated with poor sleep quality measures and more importantly with decreased motor skill acquisition in our cohort. Also, similar findings were found among healthy controls, in which subjective sleep quality and plasma serotonin levels were significantly associated with motor skill acquisition in this group.

The results of this study have demonstrated that the potential to improve performance of a new motor skill is preserved in PwPD. Understanding improvement in motor performance in PwPD has important practical implications for rehabilitation because the acquisition and reacquisition of motor skills are important parts of motor learning of functional tasks. Importantly, the findings indicated significant associations between subjective and objective sleep measures and the acquisition of the VR game in PwPD and healthy control participants. These findings suggest that impact of sleep quality on motor learning is not limited to simple motor tasks but also extends to a functional motor task that is complex and has direct implications for physical therapists. Although most previous studies that assessed the effect of sleep on motor skill learning utilized simple tasks that focus on fine motor skills (13), evidence suggests that research findings on simple motor tasks cannot be generalized to gross and more complex motor skills (20). This is evident by studies that found the effect of sleep on motor skill learning differs with task complexity (40). In this current study, participants practiced a novel functional motor task that is similar to daily activities and is often practiced in a rehabilitation setting. The VR game utilized in the current study needs gross movements. Gross motor movements require larger body segments involvement and require more complex muscle synergies (41). Therefore, these findings further shed the light on the relationship between sleep quality and performance on functional complex tasks that resembles every day activities in PwPD, and the possible impact sleep quality has on motor learning approaches commonly utilized by physical therapists in rehabilitation settings.

Previous studies reported a number of factors that might have a major impact on motor skill learning in PwPD including disease severity and duration (6, 11), and cognitive deficits (12). Our results extend this earlier research by demonstrating that individuals' sleep quality also impacts subsequent motor skill acquisition in PwPD. The findings of the current study are in line with previously published papers which indicates sleep is an important factor in motor skill acquisition in young healthy adults (42) and in people with obstructive sleep apnea (OSA) (15). Appleman et al. (14) suggested that sleep quality, assessed by actigraphy and quantified as time awake after sleep onset, is associated with subsequent motor skill acquisition. Besides, in extension to previous work investigated the importance of sleep for off-line motor learning and memory consolidation in PwPD (18), the current study found that sleep quality is also important in the online motor acquisition which is considered a very important step for motor memory formation. Bradley et al. (19) found that during the initial learning session of the motor task, the activations in a motor-related network, including the cerebellum, putamen, pallidum, and parietal cortex, forecasted subsequent offline changes in behavior. This suggests that sufficient activation in a motor-related network during motor skill acquisition is necessary to trigger sleep-facilitated consolidation in this population. Therefore, understanding factors that influence motor skill acquisition in PwPD is very important.

The current study demonstrated significant correlations between overall sleep quality as measured subjectively by PSQI and motor skill acquisition. These findings are considered important considering that PwPD have reduced sleep quality. According to PSQI scores, both groups in this study have reduced sleep quality, however, there was a significant difference in the average score of PQSI between PwPD and healthy controls. 68.1% of the PD were poor sleepers compared to 48% in the healthy control participants. These results are consistent and comparable with previous studies, which found reduced sleep quality in PwPD (16). Havlikova et al. (43) found that 73.1% of PwPD were poor sleepers during the nighttime, and the study of Menza et al. (44) demonstrated that sleep problems were very common in PD, affecting about three quarters of these individuals. In addition, the results of this current study support the age-related changes in sleep quality as around half of the healthy control participants were poor sleepers (45–47).

The current study demonstrated significant correlations between objective sleep measures (sleep efficiency and WASO) and motor skill acquisition in PwPD. Studies have confirmed the importance of having a certain amount of sleep continuity (i.e., undisrupted sleep) for motor skill learning (46). Sleep efficiency and WASO are considered important parameters of undisrupted sleep and can detect poor sleep quality especially for those suffering from insomnia (48). Improvement in sleep efficiency has become a gold standard for evaluating insomnia treatment efficacy, sleep restriction therapy (SRT), and cognitive behavioral therapy (CBT) (48). The findings expanded the previous work of Al-Sharman et al. (46) who demonstrated that continuous periods of sleep as indicated by sleep efficiency and WASO are important factors to ensure optimal sleep-dependent consolidation of a functional motor task in young healthy individuals. Also, these findings are in line with Appleman et al. (14) who indicated that WASO, significantly influenced subsequent motor skill acquisition in young healthy individuals.

In line with previous studies among PwPD and other neurological populations, the plasma serotonin levels in the current study were significantly correlated with sleep quality as measured by PSQI in PwPD. Importantly, this is the first study to examine the association between motor skill acquisition and plasma serotonin level. The findings suggest that serotonin level is significantly related to motor skill acquisition. Higher serotonin indicates better motor acquisition, since the time to complete the VR game was lower in participants with higher serotonin level. Studies reported that serotonin plays an important role in fundamental learning mechanisms and in neuroplasticity, referring that to its location in midline raphe nuclei of the brainstem and its role in regulating numerous basic functions which require energy or conserve energy (49). Furthermore, studies have confirmed the role of serotonin on the wake-sleep cycle. Several studies have indicated that serotonergic dysfunction in PwPD is associated with the development of non-motor symptoms including sleep disturbances (50). Thus, this might explain the mechanism behind this relationship (51). Interventions that might help to improve sleep quality and serotonin level might improve motor skill acquisition in PwPD. Aerobic exercise was found to improve serotonin level, and subsequently reduce pain in people with fibromyalgia (52). In multiple sclerosis, a recent study found that aerobic exercise improves sleep quality and that improvement was associated with improvements of the serotonin level (53).

This study is not without limitations. This study was performed without initial power calculations for the sample size, and accordingly the current study findings need to be interpreted cautiously. However, it should be noted that this is a pilot cross-sectional observational study. Overall, in this study, the power was found to be 97% for a sample size of 31 at a level of significance of 0.05 using the correlation coefficient with the PSQI score. Also, the power was found to be 80% for the sample size of 31 at a level of significance of 0.05 using the correlation coefficient with the plasma serotonin level. PwPD participated in this study were moderately affected by the disease [HY mean (SD) = 2.2 (0.74) units]. Replicating this study in a large cohort across the continuum of the disease is warranted. Furthermore, regarding sleep assessment, we used the subjective (PSQI), and objective (the Actisleep) to assess sleep. Both assessments are not designed to capture sleep architecture. Therefore, we cannot determine which sleep stages are associated with motor skill acquisition. Future studies are needed to use higher resolution methodologies such as polysomnography. Also, due to the limited number of Actisleep devices available in this study, only 22 PwPD wore the Actigraph which limits the interpretation of the results. These findings, however, may set the basis for future studies in this area. We assessed only serotonin as a sleep related biomarker. There are other sleep related biomarkers such as melatonin and cortisol that might affect motor skill acquisition. Future studies are required to assess these biomarkers.

The results reported here are of clinical importance considering the high prevalence of sleep disturbances in PwPD. We believe it is important to provide health care professionals mainly physical therapists with recommendations to consider sleep as an important factor affecting motor skill acquisition. It is possible to improve clinical outcomes of rehabilitation by improving sleep quality. In clinical settings, sleep assessment is not considered as a major part of a physical therapists' evaluation. However, due to the important role of sleep on learning and memory, we believe that Sleep assessment should be considered as a major part of a physical therapists' evaluation which might allow clinicians to more effectively individualize interventions to fit specific patients' characteristic to achieve maximum level of motor acquisition. In addition, it is of importance to find interventions that could improve sleep quality in this population.

## Data Availability Statement

The raw data supporting the conclusions of this article will be made available by the authors, without undue reservation.

## Ethics Statement

The studies involving human participants were reviewed and approved by Institutional Research Committees of Jordan University of Science and Technology (ID: JUST-AA-2018-525). The patients/participants provided their written informed consent to participate in this study.

## Author Contributions

AA-S, II, HK, and KE-S contributed to research idea, data collection, managing and analyzing the data, and writing and reviewing the manuscript. All authors contributed to the article and approved the submitted version.

## Conflict of Interest

The authors declare that the research was conducted in the absence of any commercial or financial relationships that could be construed as a potential conflict of interest.
